# Effect of Individual Nutrition Therapy and Exercise Regime on Gait Speed, Physical Function, Strength and Balance, Body Composition, Energy and Protein, in Injured, Vulnerable Elderly: A Multisite Randomized Controlled Trial (INTERACTIVE)

**DOI:** 10.3390/nu13093182

**Published:** 2021-09-13

**Authors:** Chad Yixian Han, Maria Crotty, Susie Thomas, Ian D. Cameron, Craig Whitehead, Susan Kurrle, Shylie Mackintosh, Michelle Miller

**Affiliations:** 1 Caring Futures Institute, College of Nursing and Health Sciences, Flinders University, Adelaide, SA 5042, Australia; chad.han@flinders.edu.au; 2 Rehabilitation and Ageing Studies Unit, Flinders University, Adelaide, SA 5042, Australia; maria.crotty@flinders.edu.au (M.C.); susan.thomas@flinders.edu.au (S.T.); craig.whitehead@rgh.sa.gov.au (C.W.); 3 John Walsh Centre for Rehabilitation Research, Faculty of Medicine and Health, University of Sydney, Sydney, NSW 2065, Australia; ian.cameron@sydney.edu.au; 4 Division of Rehabilitation and Aged Care, Hornsby Ku-ring-gai Hospital, Hornsby, NSW 2077, Australia; kurrle@bigpond.com; 5 Allied Health and Human Performance, University of South Australia, Adelaide, SA 5042, Australia; shylie.mackintosh@unisa.edu.au

**Keywords:** frail, nutrition and exercise, femur fracture, randomized controlled trial, older adults

## Abstract

It is imperative that the surgical treatment of hip fractures is followed up with rehabilitation to enhance recovery and quality of life. This randomized controlled trial aimed to determine if an individualised, combined exercise–nutrition intervention significantly improved health outcomes in older adults, after proximal femoral fracture. We commenced the community extended therapy while in hospital, within two weeks post-surgery. The primary outcome was gait speed and secondary outcomes included physical function, strength and balance, body composition, energy and protein intake. Eighty-six and 89 participants were randomized into six months individualised exercise and nutrition intervention and attention-control groups, respectively. There were no statistically significant differences in gait speed between the groups at six and 12 months. There were no major differences between groups with respect to the secondary outcomes, except estimated energy and protein intake. This may be explained by the sample size achieved. Participants in the intervention group had greater increment in energy (235 kcal; 95% CI, 95 to 375; *p* = 0.01) and protein intake (9.1 g; 95% CI, 1.5 to 16.8; *p* = 0.02), compared with those in the control group at six months but not significant at 12 months. This study has demonstrated that providing early, combined exercise and nutrition therapy can improve dietary energy and protein intake in older adults with hip fractures.

## 1. Introduction

Hip fractures are strongly associated with poor quality of life and mortality, especially in frail, older adults [1,2]. More than 30% of these older adults die within the first year of hip fracture [3,4]. This phenomenon suggests that hip fractures may be a symptom of frailty in this population, and the beginning of a downward spiral towards the end of life. Furthermore, hip fractures have significant economic impacts on healthcare systems [5,6]. The average cost per index hospitalization and at 12 months for hip fractures in older adults globally was reported to be USD$10,075 and USD$43,669 respectively [6]. Although surgery is a good option to repair hip fractures in older adults, it is imperative that such surgical treatments are followed up with rehabilitation to optimize recovery and enhance quality of life.

Rehabilitation for older adult hip-fracture patients has evolved over the years. The National Institute for Health and Clinical Excellence (NICE) guidelines recommends “[e]arly identification of individual goals for multidisciplinary rehabilitation to recover mobility and independence, and to facilitate the return of patients to their ’pre-fracture‘ residence and to long term wellbeing” [7]. Evidence suggests that intensive in-hospital [8] and outpatient/home rehabilitation [9] of post-hip-fracture surgical patients is associated with greater improvements in physical function and the activities of daily living. A recent meta-analysis identified that exercise interventions, provided in the early phase of hip-fracture rehabilitation, in older adults, can improve physical function. In addition to the timing of therapy, rehabilitation overseen by a multi-disciplinary healthcare team, after hip fracture, has been recommended to help patients return to pre-fracture living arrangements [10,11]. This multi-disciplinary support usually involves medical (i.e., orthopedic surgeons, geriatricians, rehabilitation physicians), nursing and allied health professionals (i.e., physiotherapist, occupational therapist, dietitians) [12]. Although there is evidence that the implementation of early, multidisciplinary rehabilitation benefits older adult hip-fracture patients, the type of exercise and accompanying nutritional support that should be prescribed remains unclear.

As hip-fracture patients are often malnourished [13,14], nutrition assessment and support are vital to optimization of rehabilitation exercises [11,15]. An updated Cochrane systematic review and meta-analysis in 2016 focused on nutrition supplementation for older adults with hip fracture [16]. The study reported low quality evidence to support a significant effect for oral nutrition supplements (ONS), started before or soon-after surgical intervention, to reduce risk of complication, but found no significant effect on mortality, and suggested further evaluation be conducted. Most nutrition-support studies included in this review did not individualize the prescription of ONS to each patient, but instead provided a convenient standardized amount across all participants. After this review was published, two studies have reported mixed results for nutrition interventions. Klemm and colleagues’ analyses indicated that early nutrition intervention, when delivered by dietitians, was associated with lower prevalence of malnutrition, lower incidence of pressure injuries and significant reductions in overall and subacute length of stay and incidence of pressure injury [17]. Wyers and colleagues combined nutrition counselling delivered by dietitians and ONS in a 3-month intensive nutrition intervention in older adults after hip fracture [18]. Their results showed that the intervention improved dietary intake and malnutrition status, but only for the duration of the therapy. There were no significant effects on postoperative complications or functional parameters. It was recommended that further research be done on nutrition interventions, other than ONS, to enhance clinical outcomes.

The evidence for combining both exercise and nutrition interventions to improve health outcomes in older adults with hip fracture remains unclear. In recent studies, Invernizzi and colleagues [19] found exercise and nutrition counselling (delivered by dietitians) improved physical function, while Magaziner and colleagues [20] reported that a multicomponent home-based exercise and nutrition counselling regime did not significantly improve community ambulation, defined as ability to walk 300 m or more in six minutes. The synergistic effect of combining exercise and nutrition intervention (particularly involving dietitians in its delivery) on functional outcomes, in older adults with hip fracture, is yet to be determined.

INTERACTIVE was a six-month, combined individualised exercise and nutrition therapy and program (involving physiotherapists and dietitians in its delivery), starting within two weeks of surgical intervention, with older adults after hip fracture. The aim of this randomised controlled trial (RCT) was to investigate whether the program significantly improved gait speed, physical function, strength and balance, body composition, energy and protein in older adult hip-fracture patients.

## 2. Materials and Methods

### 2.1. Study Design

This is the primary trial report of the INTERACTIVE RCT conducted from June 2007 to September 2009. In brief, the INTERACTIVE trial was a four-site RCT with blinded, assessed outcomes, 12-month follow up of community-dwelling older adults after proximal femoral fracture (PFF). The details of the study design and methods were previously published in a protocol paper [21]. The trial was registered at the Australian Clinical Trials Registry (ACTRNI2607000017426).

### 2.2. Participants and Recruitment

Older adults over 70 years, with PFF confirmed by a radiology report, were recruited from four sites in Australia: Adelaide (Flinders Medical Centre, Flinders Private Hospital, Repatriation General Hospital) and Sydney (Hornsby Ku-ring-gai Hospital). The eligibility criteria for recruitment were: ability to achieve a mini-mental state examination score (MMSE) ≥ 18/30, a body mass index (BMI) between 18.5 and 35 kg/m^2^ and residing in the community within existing local service boundaries. Exclusion criteria were: PFF was pathological or malignant, resided in residential care, unable to speak English, non-ambulatory pre-fracture, unable to tolerate any physical activity more than standing transfers post-operation or not medically stable within 14 days post-operation, as assessed by their respective primary care team. All participants provided informed consent, with additional third-party consent obtained for participants not in full capacity to do so i.e., post-operative delirium or MMSE between 18 and 23.

### 2.3. Randomization and Blinding

The process of group allocation was managed externally by the Pharmacy Department, independent to the study, at one study site. Participants were randomly assigned to either the combined-exercise-and-nutrition-therapy group or the attention-control group, using computer generated random allocation placed in sealed envelopes, after baseline measures were completed. Statistician and outcome assessors were blinded to group allocation. It was not feasible to blind therapists or participants, due to the nature of the intervention.

### 2.4. Intervention

The intervention provided is an individualised combined exercise-and-nutrition therapy that commenced during inpatient stay within two weeks post-surgery and continued for six months post-discharge to the community. The first part of the therapy involved a nutrition program delivered by a dietitian. Participants were measured with a MedGem portable indirect calorimeter [22]. This best estimate caloric requirement to prevent clinically significant weight loss and to meet individual dietary requirements, especially with respect to energy and protein. Participants had dietitian visits every two weeks (alternating with a physiotherapist), to monitor dietary fluctuations and optimize nutrition intake. The nutritional intervention used in this study had been individualised to each participant by the intervention dietitian. Strategies utilised to help achieve energy and nutrient requirements included individual counselling (timing, size, frequency of meals, recommendations of nutrient-dense foods and recipes) and referral to community meal services. Oral nutrition supplementation, protein supplements or multi-vitamins were prescribed if deemed necessary by the research dietitian. The second half of the therapy was an exercise component based on the Otago exercise program [23]. This individually tailored program has strength, balance and walking components. The strength component consists of five lower body exercises with four levels of difficulty e.g., knee extensor, with three components with the options of progressive loading with ankle weights, ranging from 1–8 kg, done three times a week, each taking approximately 30 min to complete. The balance-retraining component has nine exercises with four levels of difficulty, e.g., toe walking, three times a week, for approximately 15–20 min. The walking component can be completed as one session or as three 10-min sessions over the day. Each exercise session also begins with a 5-min gentle warm-up. Exercises were supervised until the participant was deemed safe to carry out the strength, balance and walking program independently by the research physiotherapist. Participants were asked to perform the exercises three times a week and go for walks tri-weekly on their own. The research physiotherapist visited participants every two weeks to supervise and augment the program, based on individual progress and needs. Further details of the intervention have been published in the protocol paper [21]. This exercise program has been chosen for its ease in implementation and affordability, as well as effectiveness in falls reduction in older populations [23,24,25].

### 2.5. Attention Control

Participants in the attention-control group received therapy as per standard care and respective hospital protocols i.e., continued therapy as prescribed during hospital admission (acute and rehabilitation). They also received visits by the study physiotherapist and dietitian, to match the length and frequency of social interactions received by the intervention participants. General nutrition, exercise, and information on falls prevention, which were provided to the intervention participants, were also discussed with participants in the attention-control group.

### 2.6. Primary and Secondary Outcome Measures

Baseline data collection was performed within 10 days post-surgery. The primary outcome was 3-metre gait speed, measured with a stopwatch [26]. The following methods were used to measure other physical functions, strength and balance, as secondary outcomes: physical and instrumental activities of daily living (PADL/IADL) using the Older Americans Resources and Services Program (OARS) functional assessment questionnaire [27]; knee extensor strength, using a Nicholas manual muscle tester (NMMT) [28]; grip strength on the dominant hand, using a calibrated Smedley hand dynamometer (Tokyo, Japan) [29]; and functional balance using the modified Berg Balance scale (MoBERG) [30]. The following methods were used as measures of the remaining secondary outcomes: body composition and nutrition outcomes. Percent-weight changes were measured with calibrated digital scales (BF-681 Scale and Body Fat Monitor; Tanita). Using the dual-energy X-ray absorptiometry (DXA: Lunar Prodigy, GE Healthcare, Chalfont Saint Giles, Buckinghamshire, UK), fat-free masses were measured. Dietary intakes were assessed with the 24 h dietary recall method, using a standardized protocol [31].

Quality of life was measured with the Assessment of Quality of Life (AQoL) questionnaire [32]. Results for Quality of Life together with a cost-effectiveness analysis of the intervention have been published [33]. Given the nature of the intervention, which included individual goal-setting with adaptations made in negotiation with the participant and informed by individual progress, the measurement of participant adherence to the intervention was unable to be determined using standard methodology.

### 2.7. Ethics Approval and Consent to Participate

The study was approved by the Flinders Clinical Research Ethics Committee—110/067 and the Hawkesbury Human Research Ethics Committee of the Northern Sydney Central Coast Health—07/HAWKE/21. Written consent was obtained from all participants prior to baseline assessment and randomization. A third-party consent from a close relative or immediate caregiver was sought additionally if a participant was deemed to not have the capacity to provide informed consent (i.e., post-operative delirium or MMSE between 18 and 23).

### 2.8. Sample Size

Sample size was calculated based on the gait speed data derived from a smaller similar study (*n* = 100) undertaken at one of the four RCT sites, prior to this study [34]. Using 80% power and an alpha of 0.05, it was calculated that a sample size of 176 participants in each group would be required to detect a 20% difference that would be clinically and statistically meaningful. Based on an assumed 30% drop out rate to account for deaths and withdrawals, the study aimed to recruit 460 participants (230 in each group).

### 2.9. Statistical Analysis

Participants were assessed at baseline (before randomization), six and 12 months. Data were coded to allow for blinding to group allocation during statistical analysis. Normality tests (Kolmogorov–Smirnov and Shapiro–Wilk) showed normal distribution for all measures, thus parametric tests were used. The primary outcome of gait speed was analyzed with both per-protocol (PP) and intention-to-treat (ITT) analyses principles. Independent sample t-tests and Chi-square test of association were used, as appropriate, to compare groups at baseline. Multiple imputation methods (Markov chain, Monte Carlo) were utilised to derive any missing data points, with five imputations carried out for each missing value for the ITT analyses [35]. To determine differences between the groups at six and 12 months, one-way ANCOVA and linear regression models with follow-up values as dependent variable and baseline as covariates were used. Statistical analysis was performed using SPSS for Windows version 25 (SPSS Inc, Chicago, IL, USA). Results are expressed as mean and standard deviation (SD) for continuous variables, as number (percent) for categorical variables, and differences between groups as mean difference with 95% confidence intervals (CI). Between-group Cohen D effect sizes were calculated [36].

## 3. Results

### 3.1. Recruitment

**Figure 1:**
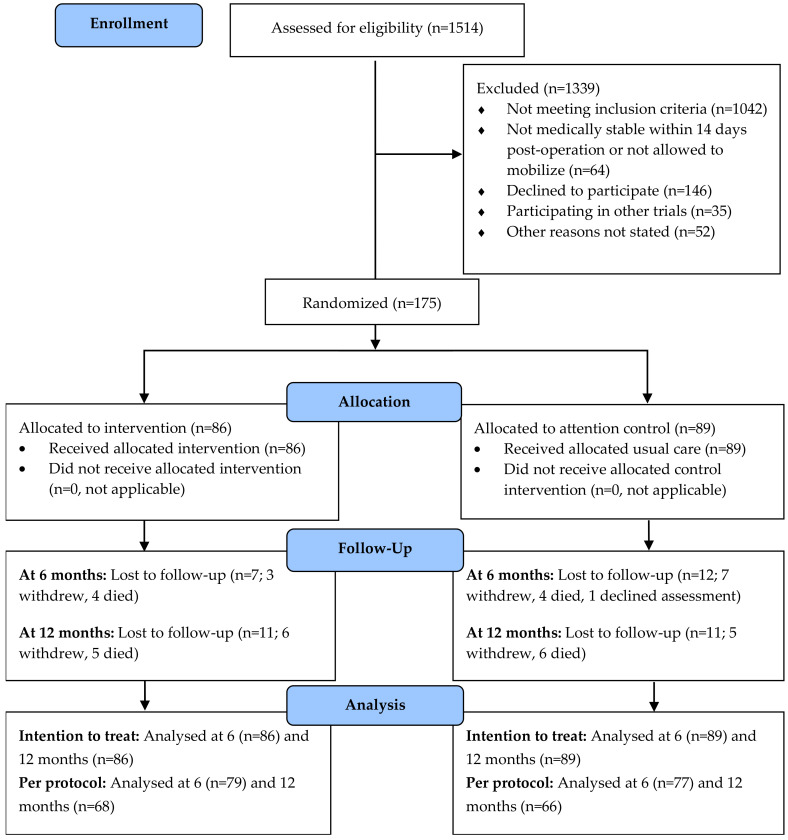
Flowchart of participants through the INTERACTIVE trial.

A total of the 1514 patients were screened consecutively at the four RCT sites over the recruitment period from June 2007 to April 2010. Due to unexpected slower recruitment rate, the desired sample size calculated was not achieved. As per Figure 1, 175 out of the 319 patients who were eligible participated in the trial. Eighty-six and 89 participants were randomized into the intervention and attention-control groups, respectively. Follow-up data from 92% (79/86) of the intervention group and 87% (77/89) of the control group were available for analysis at six months (Figure 1). At 12 months, the availability of follow-up data was 79% (68/86) and 74% (66/89) of the intervention and control groups, respectively. The follow-up attrition rate was 23.4%, with 11 of the 41 losses due to death.

### 3.2. Characteristics of the Study Population

Participant characteristics are shown in Table 1. The mean age was 82.7 years and women made up of 77.1% of the participants. Baseline characteristics between groups were well matched with the exceptions of gender and grip strength. There were more females in the control group (*p* = 0.002) and grip strength was significantly higher in the intervention group (*p* = 0.011).

### 3.3. Primary Outcome

Gait speed improved at six and 12 months in both the ITT and PP analyses, irrespective of treatment group. However, there were no statistically significant differences in gait speed between the groups at six and 12 months in either analysis. Results remain unchanged after including gender as an additional covariate.

### 3.4. Secondary Outcomes

As shown in Table 2 and Table 3, there were no major differences between groups with respect to the secondary outcomes of the trial, with exception of grip strength and estimated energy and protein intake. Overall, there were improvements in knee strength, MoBERG, PADL, IADL scores in both groups at six months and 12 months. The intervention group had higher increase in fat-free mass compared with the control group, though this difference was not statistically significant. In the PP analysis of grip strength, there was significantly better improvement in the control group as compared with the intervention group at six months. However, this improvement was not observed at 12 months follow-up or in the ITT analyses.

Participants in the intervention group had greater increment in energy intake compared with those in the control group at six months (235 kcal; 95% CI, 95 to 375; *p* = 0.01). However, this increment was not significant at 12 months in either the PP or ITT analyses. Participants in the intervention group had greater increment in protein intake compared with those in the control group at six months (9.1 g; 95% CI, 1.5 to 16.8; *p* = 0.02). Similar to energy intake, the increment in protein intake was not significant at 12 months in both PP and ITT analyses.

## 4. Discussion

There were no statistically significant differences in gait speed between the groups at six and 12 months. There were no major differences between groups with respect to the secondary outcomes, except estimated energy and protein intake. However, there were positive trends of the intervention in improving grip strength (12 months), MoBERG, PADL, IADL, fat-free mass, and significant improvements in estimated energy and protein intake, albeit not without limitations of baseline differences in grip strength and gender proportions. The lack of significant changes, with exception to energy and protein intake, may relate to a lack of statistical power. In addition, there is missing data for indirect calorimetry and body composition due to technical difficulties in obtaining these measures in this population. The benefits of the intervention may be more evident, if the desired sample size calculated was achieved. Therefore, the results presented in this study should be interpreted with caution, that it was not statistically powered, and there were differences in grip strength and proportions of females between groups at baseline.

A recent similar trial (sufficiently powered) involving multicomponent home-based physical therapy intervention in older adults with hip fracture reported a higher percentage of intervention compared with control participants, with improved walking capabilities after 16 weeks, albeit the difference was also not statistically significantly different between groups [20]. Another trial, using a 10-week home-based progressive-resistance exercise program, found significant improvement in gait speed but did not include a nutrition component [37]. There also could be other contributing factors that affected gait speed that were not measured in the present study. For example, impairments in lower body strength, perceived general health and balance confidence were identified as predictors of gait speed in older adults after hip fractures [38,39]. Potential confounders to gait speed, such as pain, were also not measured and accounted for [40]. The better improvement in grip strength seen in the control group at six months, compared with the intervention group, is likely contributed by their significant difference at baseline. The subsequent follow-up at 12 months later showed an observable trend—that the intervention group had greater improvements in grip strength at 12 months, albeit not statistically significant.

The exercise component of the present study focused on strength and balance. While effective for preventing falls [25], modifications to incorporate elements of functional exercises may also improve important aspects of rehabilitation, such as muscle fatigue and quality of life [41,42]. Functional training includes motions or exercises that use movement patterns similar to performing daily tasks and has been found to improve muscle strength, physical functioning, and the activities of daily living in older adults [43]. Moreover, implementation is equally important to the type of exercise prescribed [44], and exercise programs targeted at older adults should consider also barriers and enablers of adherence [45]. It remains a challenge to incorporate the most effective and well-adhered-to types of exercise for older adults.

The median energy and protein intakes between older men and women are different [46]. Therefore, the improvements in energy and protein intake in the present study should also be interpreted with the consideration that there were more males in the intervention compared with the control group. Nonetheless, it is worth mentioning that the baseline BMI, estimated energy and protein requirements were not significantly different despite the gender differences between groups. Furthermore, a recent meta-analysis found that there were no differences in levels of estimation in self-reported total energy intake between males and females [47]. Acknowledging the aforementioned limitation, the significantly better improvements in energy and protein intake in the intervention as compared with the control group, were consistent with previous similar studies. Nutritional care was reported to significantly increase energy and protein intake in acute hip-fracture patients [48]. Oral nutrition support provided by dietitians was previously reported to improve outcomes in older adult patients after surgical fixation of hip fractures [49]. The use of such individualised nutritional support is not novel, but still uncommon. Six out of seven RCTs from a systematic review on the effects of a geriatric team rehabilitation after hip fracture, provided no information on nutrition, with limited reports on multidisciplinary action on nutrition support [50]. In that review, only one RCT provided information that nutritional support was given in a form of a protein drink, with no individualised approach. Oral protein nutrition supplementation alone is unlikely to augment diet adequacy. A recent study of exercise and protein supplementation trial on untrained older adults, found leucine-enriched whey protein isolate did not provide additional benefits to those already having sufficient protein at baseline [51]. The authors also suggest future studies to use a whole-foods approach, to investigate if a higher protein intake is needed to alleviate muscle weakness. The individualised nutritional intervention delivered in the present study could have been better documented to allow better understanding of the effects of individualised dietary modifications beyond energy and protein i.e., diet quality, and macronutrients.

The effect of early nutritional support on Frailty, Functional Outcomes and Recovery of malnourished medical inpatients Trial (EFFORT) demonstrated that individualised nutrition support is associated with reduced adverse clinical outcomes, in medical inpatients at nutritional risk [52]. However, this effect was nullified when the same measurements were done at follow-up, suggesting the need for such individualised care to be extended beyond discharge, to the community, to sustain a “legacy effect”, as observed in pharmacotherapies [53,54]. We were unable to use a standardized method to measure adherence to therapy, given that it was individualised for each patient. Although this mimics care in usual practice, where therapy is always based on individual patient needs and not a one size fits all approach, there is a need to document and analyze dietary intakes/habits beyond any intervention period, to inform future practices. Incorporating elements of telehealth within intervention may be considered, as it can improve access to care, and in turn improve adherence [55,56].

This RCT was completed with no deviations from the published protocol. The use of an attention-control group that provided a sham intervention was one of the study’s strengths. Although it was not possible to blind treating clinicians to the interventions, the outcome assessors were blinded. As all participants received an equal number of home visits, treatment statuses were unlikely to be disclosed to the blinded assessors unless further probing was done (which was advised against during the outcome assessment). The primary outcome, gait speed, is a performance-based measure, that is less likely to be affected by observer bias. The remaining outcomes assessed were part self-reported and part performance-based. The use of a handheld indirect calorimetry also provided REE, to allow the customizing of energy needs [22]. The results presented in this study were supported with body composition analysis i.e., DEXA, which was corroborated with physical-function outcomes. The use of DEXA scans in older adults for research is not common and provides for a more accurate measurement of fat-free mass than anthropometry, which is the common substitute in research studies in this area [57]. A major limitation of the study is the lack of statistical power due to unexpected slower recruitment and missing indirect-calorimetry and body-composition data due to difficulties in obtaining these measures in this population. It was a challenge to complete data collection for many measurement outcomes for logistical reasons, i.e., immobile equipment was used for body composition, resulting in missing data. Another limitation was the lack of adherence control, and the training load of the exercise interventions could not be reported as they were not well documented.

There is a paucity of high-quality studies on combined exercise-nutrition interventions of frail older adult populations [58]. A recent review of exercise and nutrition in managing hip fracture in older adults concluded that there are still few large, long term RCTs that involve multicomponent exercise and nutrition therapy interventions [59].

## 5. Conclusions

This study found individual nutrition therapy and exercise did not significantly improve gait speed, compared with standard care in older adults with hip fractures. However, the study has demonstrated that providing early, combined exercise and nutrition therapy can improve dietary energy and protein intake. More statistically powered clinical trials should be done to determine the optimal type, dose and combination of exercise and nutrition therapy that has the most benefit on functional outcomes. Future studies should consider measuring and adjusting for known predictors to the outcomes of interest, track the type of usual care and incorporate elements of telehealth. The use of indirect calorimetry should also be considered where energy requirements and intake are of concern.

## Figures and Tables

**Table 1:** Baseline characteristics of study population.

	Intervention (*n* = 86)	Control (*n* = 89)	*p*-Value ^1^
Characteristic			
Age, years, *n*, mean ± SD	86, 82.4 ± 5.7	89, 83.0 ± 6.2	0.51
Female, *n*, (%)	86, 58 (67.4)	89, 77 (86.5)	0.002
BMI ^2^, kg/m ^2^, *n*, mean ± SD	86, 25.1 ± 3.5	89, 24.8 ± 4.2	0.67
MMSE ^3^ score, *n*, mean ± SD	59, 23.5 ± 3.2	62, 23.9 ± 3.4	0.53
Baseline resting energy expenditure (kcal/d), *n*, mean ± SD	36, 1334 ± 431	32, 1321±441	0.47
Estimated energy requirement (kcal/d), *n*, mean ± SD	86, 1734 ± 388	88, 1652±330	0.14
Estimated protein requirement (g/d), *n*, mean ± SD	86, 78.2 ± 16.9	88, 77.0 ± 15.6	0.61
Physical function, strength and balance			
Gait speed (m/s), *n*, mean ± SD	86, 0.33 ± 0.28	89, 0.28 ± 0.28	0.25
Knee strength (injured) (kg), *n*, mean ± SD	74, 6.1 ± 3.08	73, 5.2 ± 3.08	0.06
Grip strength (kg), *n*, mean ± SD	85, 18.1 ± 6.6	88, 15.7 ± 5.9	0.01
MoBERG ^4^ score, *n*, mean ± SD	85, 16.3 ± 7.8	87, 15.1 ± 8.3	0.32
Physical Activities of Daily living ^5^, *n*, mean ± SD	86, 9.63 ± 1.72	89, 9.43 ± 1.97	0.47
Instrumental Activities of Daily living ^6^, *n*, mean ± SD	86, 12.0 ± 2.0	89, 11.8 ± 2.6	0.50
Body composition, energy, and protein intake			
Reported weight loss (kg), *n* (%)	24 (13.7)	18 (10.3)	0.20
Reported amount of weight loss (kg), *n* (%)			
5 kg or less	9 (37.5)	7 (38.9)	0.99
>5 kg	12 (50)	9 (50)	
unknown	3 (12.5)	2 (4.8)	
% Fat-free mass, DEXA ^7^, *n*, mean ± SD	43, 67.4 ± 9.4	36, 66.1 ± 10.7	0.59
Estimated energy intake (kcal/d), *n*, mean ± SD	86, 1143 ± 422	88, 1125 ± 417	0.77
Estimated protein intake (g/d), *n*, mean ± SD	86, 49.5 ± 19.0	88, 47.1 ± 20.1	0.42

Data presented as number (%) or number, mean ± standard deviation. ^1^ Chi-square, independent samples t-test as appropriate. ^2^ Body Mass Index. ^3^ Mini mental state examinations. ^4^ Modified Berg Balance Scale. ^5^ Physical Activities of Daily Living using the Older Americans Resources and Services Program functional assessment questionnaire, range from 0–14. ^6^ Instrumental Activities of Daily Living using the Older Americans Resources and Services Program functional assessment questionnaire, range from 0–14. ^7^ Dual-energy X-ray absorptiometry.

**Table 2:** Effects of intervention on primary and secondary outcomes, per protocol analyses.

	Intervention Group	Control Group	Mean Difference between Groups ^1^	*p*-Value ^2^	*Cohen D*
Primary outcomes					
Gait speed (m/s),					
6-month, *n*, mean ± SD	77, 0.83 ± 0.3	76, 0.83 ± 0.3	−0.02 (−0.11 to 0.07)	0.64	0.18
12-month, *n*, mean ± SD	65, 0.92 ± 0.4	67, 0.84 ± 0.3	0.08 (−0.04 to 0.21)	0.19	0.11
Change from 0 to 6-month	77, 0.49 ± 0.32	76, 0.53 ± 0.32			
Change from 0 to 12-month	65, 0.60 ± 0.47	67 0.57 ± 0.44			
Secondary outcomes—Physical function, strength and balance					
Knee strength (injured) (kg)					
6-month, *n*, mean ± SD	74, 10.7 ± 3.7	73, 10.7 ± 4.7	−0.15 (−1.59 to 1.29)	0.84	0.39
12-month, *n*, mean ± SD	63, 11.1 ± 5.1	63, 11.1 ± 5.1	0.12 (−1.99 to 1.76)	0.9	0.29
Change from 0 to 6-month	58, 4.77 ± 3.90	65, 5.43 ± 4.54			
Change from 0 to 12-month	52, 5.32 ± 5.21	55, 5.82 ± 5.15			
Grip strength (kg)					
6-month, *n*, mean ± SD	79, 18.7 ± 7.6	76, 17.7 ± 6.4	−1.24 (−2.29 to −0.18)	0.02	0.21
12-month, *n*, mean ± SD	64, 19.4 ± 8.3	67, 17 ± 5.7	2.36 (−0.15 to 4.88)	0.07	0
Change from 0 to 6-month	79, 0.57 ± 3.61	76, 1.85 ± 2.81			
Change from 0 to 12-month	64, 0.82 ± 10.85	67, 0.67 ± 8.11			
MoBERG ^3^ score					
6-month, *n*, mean ± SD	78, 38.2 ± 11.9	74, 37.2 ± 10.9	0.77 (−2.46 to 4.01)	0.64	0.03
12-month, *n*, mean ± SD	63, 39.1 ± 12.6	66, 36.5 ± 11.8	3.04 (−1.30 to 7.38)	0.17	0.17
Change from 0 to 6-month	78, 22.13 ± 10.86	73, 21.57 ± 9.81			
Change from 0 to 12-month	62, 23.6 ± 15.4	64, 21.8 ± 14.6			
PADL ^4^ score					
6-month, *n*, mean ± SD	79, 12.6 ± 1.54	78, 12.6 ± 1.86	−0.05 (−0.53 to 0.43)	0.84	0.11
12-month, *n*, mean ± SD	65, 10.98 ± 1.46	68, 10.85 ± 1.22	0.12 (−0.34 to 0.58)	0.6	0.04
Change from 0 to 6-month	79, 3.06 ± 1.87	78, 3.18 ± 1.81			
Change from 0 to 12-month	65, 1.51 ± 2.19	68, 1.51 ± 2.28			
IADL ^5^ score					
6-month, *n*, mean ± SD	79, 10.9 ± 2.8	78, 10.8 ± 3.4	−0.09 (−0.83 to 0.66)	0.82	0.04
12-month, *n*, mean ± SD	65, 11.4 ± 2.9	68, 10.8 ± 3	0.51 (−0.52−1.54)	0.33	0.17
Change from 0 to 6-month	79, −1.24 ± 2.2	78, −1.14 ± 2.48			
Change from 0 to 12-month	65, −0.85 ± 3.55	68, −0.88 ± 3.83			
Secondary outcomes—Body composition, energy and protein intake					
% Fat-free mass ^6^					
6-month, *n*, mean ± SD	33, 69 ± 9.7	36, 64 ± 10.3	1.28 (−1.53 to 4.10)	0.37	0.37
12-month, *n*, mean ± SD	41, 67.5 ± 10.4	34, 65.7 ± 10.6	−0.53 (−3.51 to 2.45)	0.72	0.05
Change from 0 to 6-month	29, 0.39 ± 5.47	37, −0.95 ± 5.96			
Change from 0 to 12-month	25, −0.02 ± 5.87	30, 0.65 ± 5.51			
Estimated energy intake (kcal/d)					
6-month, *n*, mean ± SD	79, 1701 ± 466	75, 1444 ± 444	235 (95 to 375)	0.01	0.57
12-month, *n*, mean ± SD	64, 1634 ± 436	67, 1620 ± 544	22 (−153 to 196)	0.81	0.01
Change from 0 to 6-month	76, 563 ± 578	72, 294 ± 509			
Change from 0 to 12-month	61, 480 ± 597	64, 503 ± 643			
Estimated protein Intake (g/d)					
6-month, *n*, mean ± SD	79, 67.4 ± 25.8	75, 55.8 ± 25.2	9.1 (1.50 to 16.8)	0.02	0.47
12-month, *n*, mean ± SD	64, 65.1 ± 23.6	67, 59.5 ± 20.8	6.1 (−1.76 to 14.0)	0.13	0.16
Change from 0 to 6-month	76, 16.95 ± 29.5	72, 7.93 ± 26.9			
Change from 0 to 12-month	61, 14.8 ± 30.3	64, 11.6 ± 27.8			

Data presented as number (%) or number, mean ± standard deviation. ^1^ Mean differences from one-way ANCOVA with follow-up values as a dependent variable and baseline values as a covariate. ^2^ *p*-values, derived from one-way ANCOVA with baseline values as a covariate, are for the differences in mean between intervention and control group. ^3^ Modified Berg Balance Scale. ^4^ Physical Activities of Daily Living using the Older Americans Resources and Services Program functional assessment questionnaire, range from 0–14. ^5^ Instrumental Activities of Daily Living using the Older Americans Resources and Services Program functional assessment questionnaire, range from 0–14. ^6^ Fat-free mass measured with dual-energy X-ray absorptiometry.

**Table 3:** Effects of intervention on primary and secondary outcomes, intention to treat analyses.

	Intervention Group (*n* = 86)	Control Group (*n* = 89)	Mean Difference between Groups ^1^	*p*-Value ^2^	Cohen D
Primary outcomes					
Gait speed (m/s)					
6-month, mean ± SD	0.82 ± 0.04	0.81 ± 0.04	−0.02 (−0.11 to 0.08)	0.72	0.14
12-month, mean ± SD	0.69 ± 0.04	0.66 ± 0.04	0.07 (−0.06 to 0.19)	0.36	0.07
Change from 0 to 6-month	0.49 ± 0.04	0.53 ± 0.04			
Change from 0 to 12-month	0.58 ± 0.07	0.56 ± 0.06			
Secondary outcomes–Physical function, strength and balance					
Knee strength (injured) (kg)					
6-month, mean ± SD	10.4 ± 0.45	10.3 ± 0.54	−0.12 (−1.34 to 1.10)	0.85	0.26
12-month, mean ± SD	10.9 ± 5.04	11.0 ± 5.65	−0.34 (−1.96 to 1.27)	0.7	0.26
Change from 0 to 6-month	4.77 ± 0.50	5.17 ± 0.50			
Change from 0 to 12-month	4.93 ± 0.76	5.68 ± 0.66			
Grip strength (kg)					
6-month, mean ± SD	18.8 ± 0.83	17.3 ± 0.68	−0.90 (−1.92 to 0.13)	0.1	0.14
12-month, mean ± SD	18.8 ± 0.99	17.3 ± 0.77	0.19 (−0.70 to 3.90)	0.19	0.14
Change from 0 to 6-month	0.65 ± 0.44	1.62 ± 0.35			
Change from 0 to 12-month	0.72 ± 1.24	1.68 ± 0.99			
MoBERG ^3^ score					
6-month, mean ± SD	38.0 ± 1.3	36.8 ± 1.3	0.40 (−2.81 to 3.62)	0.75	0
12-month, mean ± SD	39.3 ± 1.77	37.2 ± 1.46	2.23 (−1.56 to 6.02)	0.32	0.11
Change from 0 to 6-month	21.71 ± 1.23	21.7 ± 1.25			
Change from 0 to 12-month	22.99 ± 1.97	22.11 ± 1.76			
PADL ^4^ score					
6-month, mean ± SD	12.6 ± 0.18	12.6 ± 0.20	−0.09 (−0.55 to 0.37)	0.72	0.11
12-month, mean ± SD	11.1 ± 0.19	10.9 ± 0.17	0.49 (−0.25 to 0.62)	0.49	0
Change from 0 to 6-month	3.0 ± 0.22	3.2 ± 0.2			
Change from 0 to 12-month	1.52 ± 0.26	1.52 ± 0.26			
IADL ^5^ score					
6-month, mean ± SD	10.9 ± 0.31	10.62 ± 0.37	0.78 (−0.63 to 0.79)	0.76	0.03
12-month, mean ± SD	11.4 ± 0.4	10.9 ± 0.38	0.48 (−0.49 to 1.44)	0.38	0.03
Change from 0 to 6-month	−1.12 ± 0.26	−1.17 ± 0.28			
Change from 0 to 12-month	−0.64 ± 0.46	−0.91 ± 0.44			
Secondary outcomes–Body composition, energy and protein intake					
% Fat-free mass ^6^					
6-month, mean ± SD	68.1 ± 2.4	67.1 ± 2.1	1.07 (−1.71 to 3.86)	0.44	0.03
12-month, mean ± SD	67.9 ± 3.7	68.7 ± 3.7	−0.71 (0.47 to 0.96)	0.51	0.21
Change from 0 to 6-month	1.0 ± 1.5	−0.13 ± 1.13			
Change from 0 to 12-month	0.84 ± 2.01	1.49 ± 2.63			
Estimated energy intake (kcal/d)					
6-month, mean ± SD	1689 ± 293	1440 ± 58	240 (101 to379)	0.01	0.55
12-month, mean ± SD	1655 ± 63	1630 ± 81	20 (−136 to 177)	0.68	0.02
Change from 0 to 6-month	526 ± 70	320 ± 67			
Change from 0 to 12-month	493 ± 82	511±89			
Estimated protein intake (g/d)					
6-month, mean ± SD	66.7 ± 3.1	55.4 ± 3.0	10.54 (2.73 to 18.36)	0.01	0.45
12-month, mean ± SD	64.1 ± 3.4	59.9 ± 2.9	4.09 (−3.02 to 11.20)	0.29	0.09
Change from 0 to 6-month	16.7 ± 3.62	8.31 ± 3.37			
Change from 0 to 12-month	14.1 ± 4.09	12.8 ± 3.36			

Data presented as mean ± standard deviation. ^1^ Mean differences from one-way ANCOVA with follow-up values as a dependent variable and baseline values as covariate. ^2^ *p*-values, derived from one-way ANCOVA with baseline values as a covariate, are for the differences in mean between intervention and control group. ^3^ Modified Berg Balance Scale. ^4^ Physical Activities of Daily Living using the Older Americans Resources and Services Program functional assessment questionnaire, range from 0–14. ^5^ Instrumental Activities of Daily Living using the Older Americans Resources and Services Program functional assessment questionnaire, range from 0–14. ^6^ Fat-free mass measured with Dual-energy X-ray absorptiometry.

## Data Availability

The data presented in this study are not available due to intellectual property rights.
